# Whooping Cough

**Published:** 1885-11

**Authors:** 


### Whooping Cough.

| | | |
|---|---|---|
| Ammoniac | $1\frac{1}{2}$ to $7\frac{1}{2}$ | grains. |
| Syrup of orange-flowers or of red poppy, | 375 | " |
| Infusion of elecampane or of Virginia snakeroot | 1,125 | " |

Dose, a teaspoonful, to be repeated with greater or lesser frequency, according to the child's age and the effect produced. When the expectoration is very abundant and as if formed of muco-pus, a terebinthinate (sirup of fir-cones, of eucalyptus, or of turpentine) is prescribed. Flowers of sulphur, mixed with honey (from three-quarters of a grain to two grains or more of sulphur), may also be given twice a day. It is well, too, to rub the chest or the sides of the neck with a soothing ointment, such as one containing a drachm of the extract of aconite or of conium to half an ounce of lard.—*N. Y. Medical Journal*.

